# Nanoindentation and Hierarchy Structure of the Bovine Hoof Wall

**DOI:** 10.3390/ma14020289

**Published:** 2021-01-08

**Authors:** Bingfeng Wang, Yiyu Huang, Bingqing Zhou, Wenshu Li, Haoyu Chen

**Affiliations:** 1 School of Materials Science and Engineering, Central South University, Changsha 410083, China; huangyiyu2020@hotmail.com (Y.H.); zhoubingqing2020@outlook.com (B.Z.); liwenshu2020@hotmail.com (W.L.); chenhaoyujxt@hotmail.com (H.C.); 2 State Key Laboratory for Powder Metallurgy, Central South University, Changsha 410083, China

**Keywords:** mechanical property, biological materials, 3D printing, microstructure

## Abstract

The bovine hoof wall with an α-keratin structure protects the bovine foot from impact loads when the cattle are running. Reduced modulus, hardness and creep behavior of the bovine hoof wall have been investigated by a nanoindentation technique. The average reduced modulus of the Transverse Direction (TD) specimens from the outside to inside wall is 3.76 and 2.05 GPa, respectively, while the average reduced modulus of the Longitudinal Direction (LD) specimens from the outside to inside wall is 4.54 and 3.22 GPa, respectively. Obviously, the orientation and the position of the bovine hoof wall have a significant influence on its mechanical properties. The use of the generalized Voigt–Kelvin model can make a good prediction of creep stage. Mechanical properties of the LD specimens are stronger than those of the TD specimens. The bovine hoof wall has a layered structure, which can effectively absorb the energy released by the crack propagation and passivate the crack tip. Therefore, a kind of structural model was designed and fabricated by three-dimensional printing technology, which has a 55% performance improvement on fracture toughness. It is believed that the reported results can be useful in the design of new bionic structure materials which may be used in motorcycle helmets and athletes’ protective equipment to achieve light weight and improved strength at the same time.

## 1. Introduction

Biomaterials have created a variety of amazing functions through billions of years of evolution, such as: optimized multi-scale, hierarchical structures, excellent mechanical properties, adaptability and self-healing ability [1,2]. These biomaterials with special functions and structures can provide inventors with miraculous inspiration. Moreover, most biomaterials are essentially complex viscoelastic composites and their mechanical properties are based on various factors, e.g., details of the internal hierarchical structure, composition and water content [3].

The bovine hoof wall is a noteworthy example. Researchers have proved that the main constituents of the hoof wall are α-keratin and keratinized cells [4]. This is very interesting, because the keratin mainly contains organic proteins, rather than the other stronger biological materials compositing with high mineral content, e.g., teeth. Not only can it support the heavy body and provide physical protection, but it also has a strong passing ability: resistance to wear against the ground. It is necessary to study the relationship between the mechanical properties and the hierarchical structure because of the irreparability once it is keratinized [5,6].

Nanoindentation technology is a set of test systems for micromechanical properties of materials, which is widely used for the characterization of surface properties of film materials, biomaterials and so on. It can obtain various mechanical properties such as hardness, elastic modulus and strength [6]. Up until now, many kinds of biomaterials have been measured at the micro/nano scale. Huang et al. [7] carried out nanoindentation tests on the equine hoof wall and obtained the characterization of the hierarchical structure as well as the energy dissipation mechanisms under different water content and strain states. Hang et al. [8] used atomic force microscopy to test the tensile strength of a single fiber of the antler collagen. The transverse stiffness of the equine hoof is 10–40% less than that of the longitudinal stiffness [9,10]. The elastic modulus of the ostrich claw along the length direction is 1.84 GPa, while that in the vertical direction is 1.33 GPa. Anisotropic properties of the equine hoof and ostrich claw are weaker than that of some keratin materials. For example, the transverse stiffness of porcupine hair is only 10% of the longitudinal stiffness, while that of horse hair is 5%. This is because the orientation of the keratin fibers of porcupine hair and horse hair are parallel to the axis. In the state of water content, the stiffness of the fiber is much greater than that of the matrix, so the longitudinal stiffness becomes greater due to the reinforcement of the fibers. Additionally, dynamic nanoindentation has been applied to determine the dynamic properties of polymeric materials [11,12], bone [13,14,15], nacre [16] and soft tissue [17].

Melt deposition technology is a mature 3D printing technology for polymer materials. Under the control of a computer, the nozzle of the printer heats the filamentous raw material to a temperature slightly higher than its melting point and deposits it on the printing platform. The nozzle then moves on the X-Y plane. With the movement of the printing platform on the Z axis and the continuous extrusion of the material, the designed models have been established [18]. The same photo-curing technology is also a common additive manufacturing technology for polymer materials, and its product precision may be higher than melt deposition technology [19].

Nowadays, biomimetic technology is becoming a kind of practical and popular method, providing novel ideas for new designs. Three-dimensional (3D) printing technology is a great choice; it can not only achieve any irregular geometric shape with a complex architecture [20], but also can print biological tissue with bioink [21] and multiple materials [22] at the same time. Thus, the use of biomimetic design principles and 3D printing technology has the capacity to develop new structural and functional materials. For example, caseous calcification of the mitral annulus is a dangerous disease. In the process of overcoming this disease, it has been found that 3D-printed materials are effective in transcatheter mitral valve replacement [23,24]. In addition, in the field of bone repair, 3D printing technology is outstanding; although using traditional materials for bone repair can be a complex process, it can achieve good results. The use of 3D printing technology can also simplify the production process. Furthermore, the bone repair material can be designed as a porous material, which not only shows appropriate biocompatibility, but also a Young’s modulus that is closer to the original bone [25,26].

This work aims to study the microstructure and viscoelastic mechanical properties of the bovine hoof wall, investigate the creep process of the bovine hoof wall, propose a kind of bionic structure of the bovine hoof wall and fabricate the bionic structure through the 3D printing technique.

## 2. Materials and Methods

### 2.1. Specimen Preparation

The fresh bovine hooves were acquired from a local slaughterhouse (Changsha, China). The hooves were checked for any visible pathological damage and were carefully washed and disinfected before being stored in a freezer at −10 °C. Each specimen was carefully cut and protected.

Parry et al. [27] found that the performance of the part near the growth line of the hoof wall was better than that of the other parts, so this part was selected as the test material for nanoindentation. The nanoindentation specimens were selected from the near growth line part of the hoof wall and were divided into Transverse-Direction (TD) and Longitudinal-Direction (LD) specimens depending on the tubular axis, which is along the growth line. The nanoindentation specimens (each with a diameter of 18 mm and a height of 7 mm, which were made of the bovine hoof wall) were coated with epoxy resin. After 10 passes of water abrasive paper (400–5000 mesh) polishing, the surface of the specimen met the requirements of the nanoindentation test.

### 2.2. Plane Strain Fracture Toughness Test

In this experiment, the American Society of Testing Materials (ASTM) E1820-18 [28] test standard was fully followed. The testing equipment was an Instron-3369 (Instron, Norwood, MA, USA) electronic universal mechanical testing machine with a tensile rate of 2 mm/min. The process was described in detail in our previous paper [29].

### 2.3. Nanoindentaion Test

The equipment used in this study also included the Nano Test Vantage (Micro Materials Limited, Wrexham, UK), which was equipped with a Berkovich diamond indenter. The nanoindentation instrument was controlled by an electromagnetic drive loading system with a high-precision coil and a permanent magnet. The loading force and displacement were less than 500 mN and 20 μm, respectively. The load resolution and displacement resolution were 3 nN and 0.002 nm, respectively.

**Figure 1:**
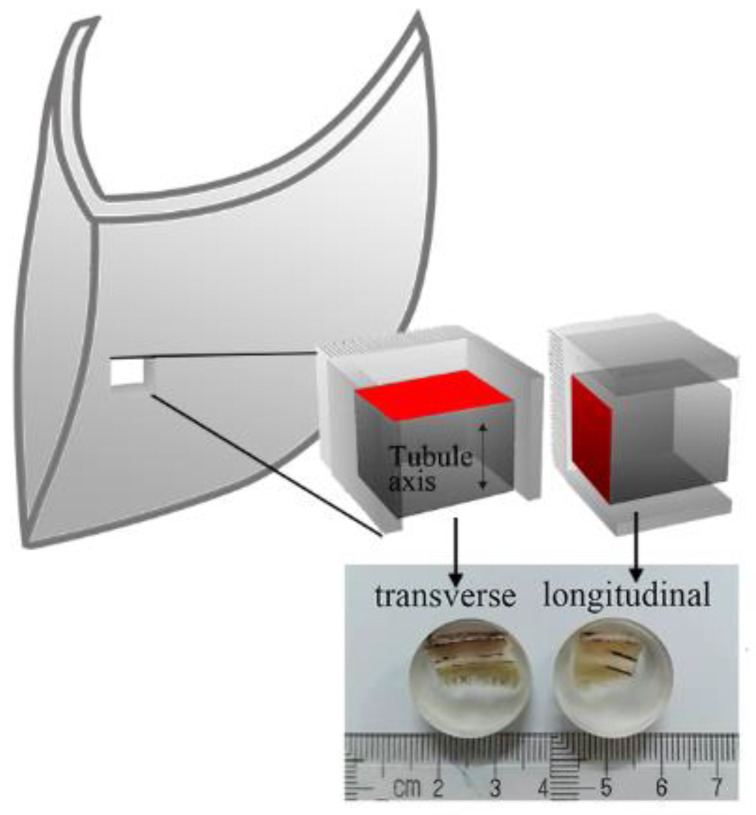
Schematic diagram of the nanoindentation specimens’ preparation.

In this experiment, the mechanical properties and microstructure of two orientations and three regions of the bovine hoof wall were tested. The maximum load, the stain rate and the holding time were 20 mN, 1 mN/s and 15 s, respectively. A series of viscoelasticity tests with strain rates of 1, 2, 5 and 10 mN/s were conducted. The specimens is shown in Figure 1.

### 2.4. 3D Printing

A kind of bionic structural model of the bovine hoof wall was designed and fabricated by 3D printing technology. The printing samples were designed in CAD Software (SolidWorks 2017 SP4.1, USA).

The 3D printer used was a CR-5S (Creality3D^®^, Shenzhen, China) and the printing material was polylactic acid (PLA, Creality3D^®^, China). The density and diameter of the filament material were 1.25 ± 0.05 g/cm^3^ and 3 mm, respectively. The melt flow rate was 0.7 g/min at 190 °C. The printing accuracy in every direction was 100 μm. The nozzle diameter was 0.4 mm, and the nozzle temperature and printing platform temperature were 200 and 50 °C, respectively. The filling ratios of all samples were 100%, and they were all printed in the same direction.

### 2.5. Microscopic Observation

In this experiment, a Quanta200 (FEI, made in Hillsboro, OR, USA) scanning electron microscope was used. Under the working voltage of 10 kV, the microstructure and morphology of the fracture area and the microtubules around the fracture area of the bovine hoof (coated with conductive coating) were observed.

## 3. Results

### 3.1. Nanoindentation of the Bovine Hoof Wall

**Figure 2:**
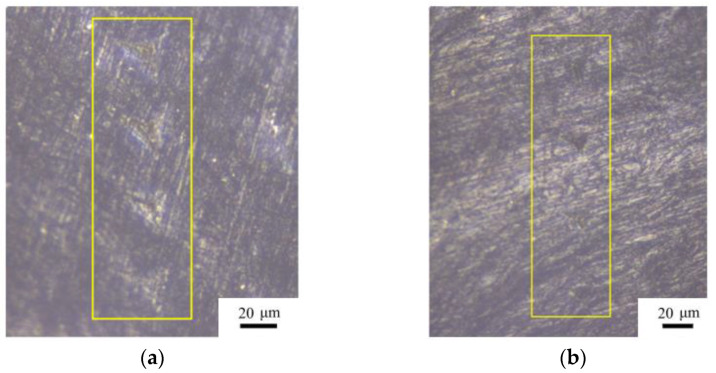
Optical micrographs of indentations in the bovine hoof wall. (**a**) Partial indentation points of the Transverse-Direction (TD) specimen, (**b**) partial indentation points of the Longitudinal-Direction (LD) specimen.

Figure 2a,b show the indentation regions of the TD and LD specimens, respectively. The indentation regions are marked by the yellow boxes. Under the same force, the areas of the indentation of the TD specimen are larger than those of the LD specimen, which indicates that the surface of the TD specimen has a lower hardness.

**Figure 3:**
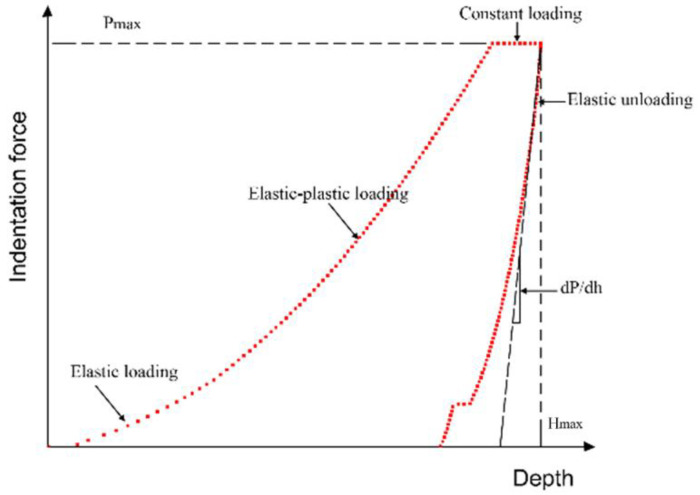
The typical indentation load–depth (P-h) curve.

Figure 3 shows a typical nanoindentation load displacement P-h curve. The maximum indentation load (*P*_max_) and the maximum indentation depth (*H*_max_) can be obtained from the curve. The elastic modulus of the material can be estimated by the elastic unloading stiffness (*E*), commonly known as reduced modulus (*E*_r_).

The nanoindentation theory of materials has been widely discussed by researchers [30,31,32,33,34,35], and the determination of modulus and hardness by the Oliver–Pharr method has been widely accepted [32]. The reduced modulus (*E*_r_) and hardness (*H*) can be derived from the following equations [33].

(1) $$\frac{1}{E_{r}}={\left(\frac{1-\nu^{2}}{E}\right)}_{\mathrm{specimen}}+{\left(\frac{1+\nu^{2}}{E}\right)}_{\mathrm{indenter}}$$

(2) $$E_{r}=\frac{\sqrt{\pi }}{2\beta }\frac{S}{\sqrt{A\left(h_{c}\right)}}$$

(3) $$H=\frac{P_{\max}}{A\left(h_{c}\right)}$$

where *E* and *ν* are the elastic modulus and the Poisson’s ratio of the specimen and the indenter, respectively. For the diamond indenter, values of *E* and *ν* are equal to 1114 GPa and 0.07, respectively [34]. *A*(*h*_c_) is the contact area produced by the indenter and specimen. β is the geometric constant of the indenter, which is equal to 1.034 [35] for the Berkovich indenter.

**Figure 4:**
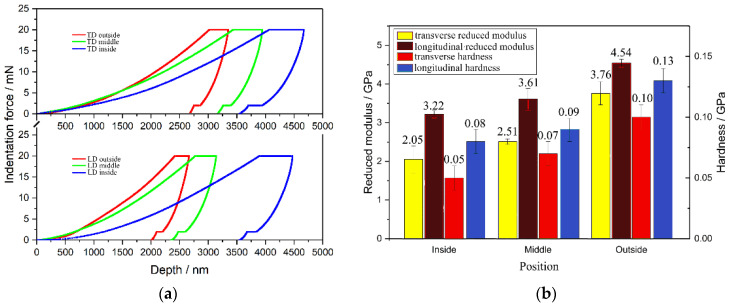
Mechanical properties of the bovine hoof wall specimens. (**a**) P-h curves for the TD and LD specimens; (**b**) Reduced modulus and Hardness in different positions in the bovine hoof wall specimens.

Figure 4 shows mechanical properties of the bovine hoof wall in different regions. Figure 4a shows the P-h curves of different regions. It can be seen that the indentation depth of the same position of the TD specimen is much greater than that of the LD specimen, indicating that the rigidity of the LD specimen is higher than that of the TD specimen. Figure 4b shows a comparison of the mechanical properties of the bovine hoof wall. From outside to inside, values of the *E*_r_ and *H* of the wall are decreased. The average values of the *E*_r_ and *H* of the outer wall of the LD specimen are 4.54 and 0.13 GPa, respectively, while the average values of the *E*_r_ and *H* of the outer wall of the TD specimen are 3.76 and 0.10 GPa, respectively. Furthermore, the mechanical properties of the LD specimen in the same part have a greater improvement compared with the TD specimen. Therefore, the LD specimen has a higher reduced modulus and hardness than the TD specimen, the external parts have a greater reduced modulus and hardness than the internal parts, and the outside parts of the bovine hoof wall in LD specimens have the best reduced modulus and hardness.

**Figure 5:**
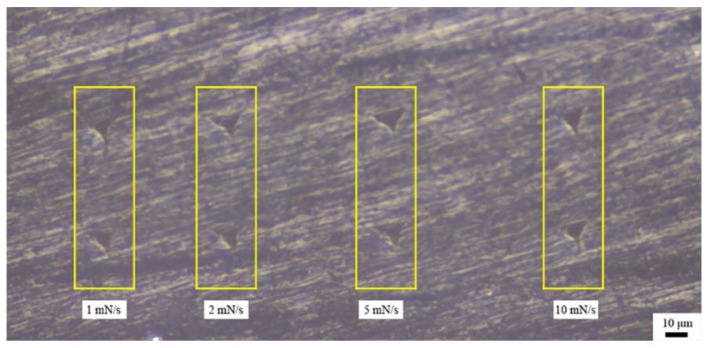
Optical micrograph of the indentation tested with different strain rates.

Nanoindentation tests with different strain rates were carried out in the middle region of the LD specimen. The strain rates were 1, 2, 5 and 10 mN/s. Figure 5 shows the optical micrograph of the indentation region of the LD specimen under four levels of strain rates, where clearly visible triangular conical indentation regions are observed. The indentation depth increases significantly with the strain rate.

**Figure 6:**
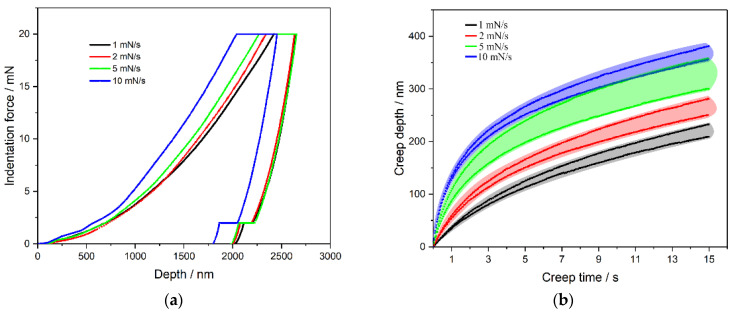
Effect of strain rate on mechanical properties. (**a**) P-h curves under different strain rates, (**b**) creep curves under different strain rates.

Figure 6 shows the effect of the strain rates on the mechanical properties of the bovine hoof wall. Figure 6a shows the P-h curves of the nanoindentation process. It can be seen that the responses of different strain rates were quite different before the pressure-maintaining stage. For viscoelastic materials, a higher strain rate leads to a greater stress lagging phenomenon; i.e., a higher strain rate produces a shallower indentation depth. Figure 6b corresponds to the pressure-maintaining stage, where the stress lagging phenomenon is relieved and a higher strain rate gets a deeper creep depth. When the pressure-unloading stage is carried out, different strain rates basically obtain a similar indentation depth. Therefore, mechanical properties of the bovine hoof wall are sensitive to the strain rate.

### 3.2. Morphology and Microstructure

**Figure 7:**
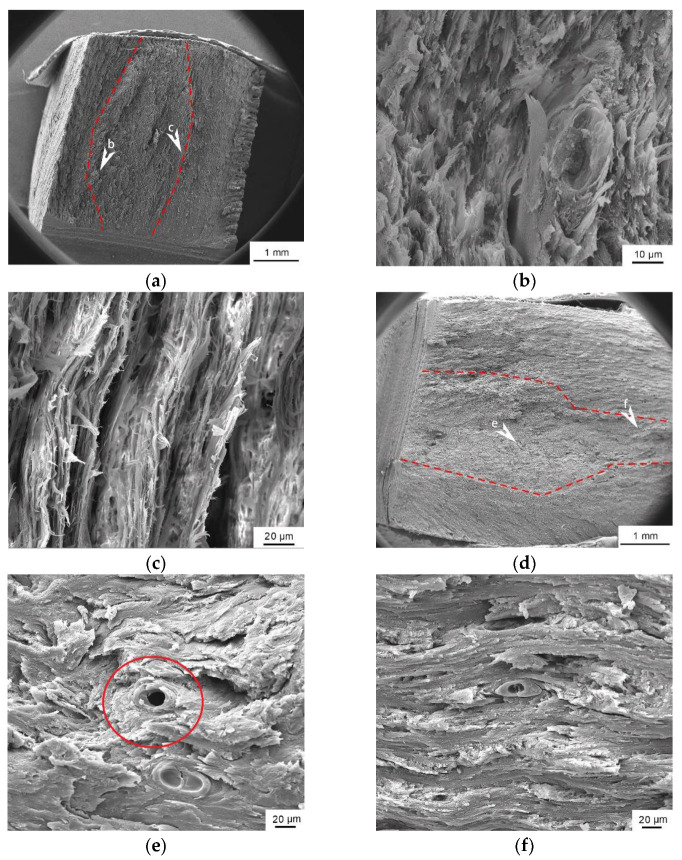
Scanning electron micrographs of the bovine hoof wall. (**a**) Scanning electron micrograph of the longitudinal specimen. (**b**,**c**) are the enlarged views marked in (**a**). (**d**) Scanning electron micrograph of the transverse specimen. (**e**,**f**) are the enlarged views marked in (**d**).

Figure 7 shows the fracture sectional view of the bovine hoof wall. There are three kinds of fracture regions marked by the red dashed lines in Figure 7a,d; Figure 7a–c show the scanning electron micrographs of the LD specimen, in which the clear river-like patterns of the lamellar structure can be seen. However, there are not only lamellar structures, but also tubular structures in the TD specimen, as shown in Figure 7d–f. Tubules with a medullary cavity are marked by the red circle in Figure 7e. Figure 7f shows the lamellar structure with severe deformation.

**Figure 8:**
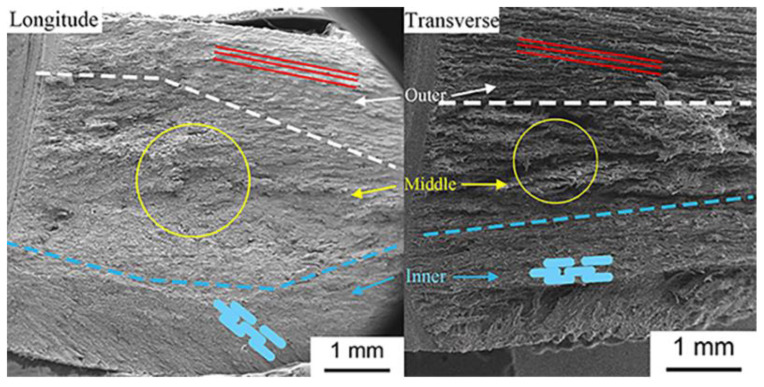
Scanned electron diagrams of the fracture face.

Figure 8 shows the scanned electron diagrams of the fracture face. The TD and LD specimens all show three fracture modes from the outside to the inside. Layered structures can be seen in the outside of the hoof wall, and the outer layer with this structure can form multiple interlocking interfaces that have a similar effect to a multi-stage deceleration structure, i.e., it can effectively absorb the energy released by crack propagation and passivate the crack tip. In the middle layer, there are some irregular bulges and depressions along the crack propagation direction, which are the manifestation of serious deformation and fracture of the fiber material. This has the effect of dispersing stress and absorbing energy. In the inner layer, the fracture is very smooth. This hierarchical structure shows that different layers have their own special functions—and the main effect of this layer is protecting the internal tissues.

## 4. Discussion

### 4.1. Analysis of Creep Property of the Bovine Hoof Wall

**Figure 9:**
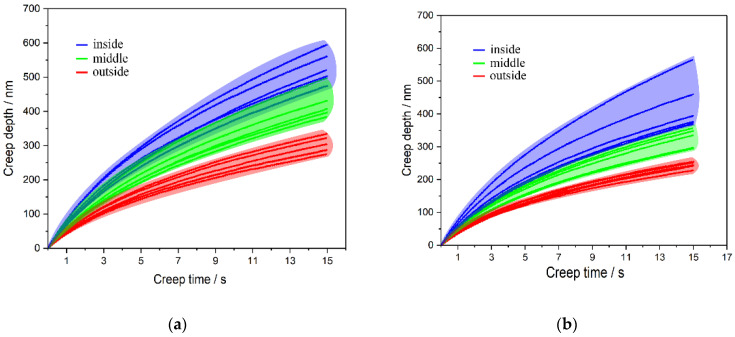
Creep curves of the bovine hoof wall. (**a**) For the transverse specimen (TD), (**b**) For the longitudinal specimen (LD).

The creep behavior of the material shows the phenomenon of mechanical relaxation. Figure 9 shows the curves of creep displacement and pressure-maintaining time in different orientations and positions of the bovine hoof wall. The different orientations of the bovine hoof wall show a similar trend, in that the creep depth gradually decreases from the inside to the outside wall of the pressure-maintaining creep stage. That is to say, the material shows a decrease in the resistance of plastic deformation from the outside to the inside wall.

It has been proposed that a combination with the ideal spring and dashpot in various methods can simulate the pressure-relaxation process of polymer materials. An advantage of this method is that it can obtain various intuitive mathematical expressions. The most commonly used and easily understood model is the Voigt–Kelvin model. The Voigt–Kelvin model is composed of the parallel connection of ideal spring and dashpot, and the stress is shared by all components while the strain of each component remains the same. The generalized Voigt–Kelvin model is used in this paper, as follows.

(4) $$J\left(t\right)=C_{0}+C_{v}t+\sum C_{j}\left[1-exp(-t/\tau_{j})\right]$$

where *C*_0_ and *C*_j_ are the compliance constant, *C*_v_ represents the compliance of the dashpot and τ_j_ is the time constant (relaxation time).

The fitting parameter of the generalized Voigt–Kelvin model is shown in Table 1. The fitting results show that the determination coefficient *R*^2^ of the fitting curves are larger than 0.99, which shows that the generalized Voigt–Kelvin model can predict the creep mechanical properties of the bovine hoof wall well.

### 4.2. Bionic Design of the Bovine Hoof Wall

In this work, nanoindentation results show that the LD specimen has higher reduced modulus and hardness than the TD specimen, the external parts have greater reduced modulus and hardness than the internal parts, and the outside parts of the bovine hoof wall in LD specimens have the best reduced modulus and hardness. Our previous work [28] also showed that the fracture strength of LD specimens is higher than that of TD specimens with the same moisture content. Therefore, a structural model of the hoof wall was proposed to clarify the relationship between the morphology and the crack propagation mechanism of the hoof wall.

**Figure 10:**
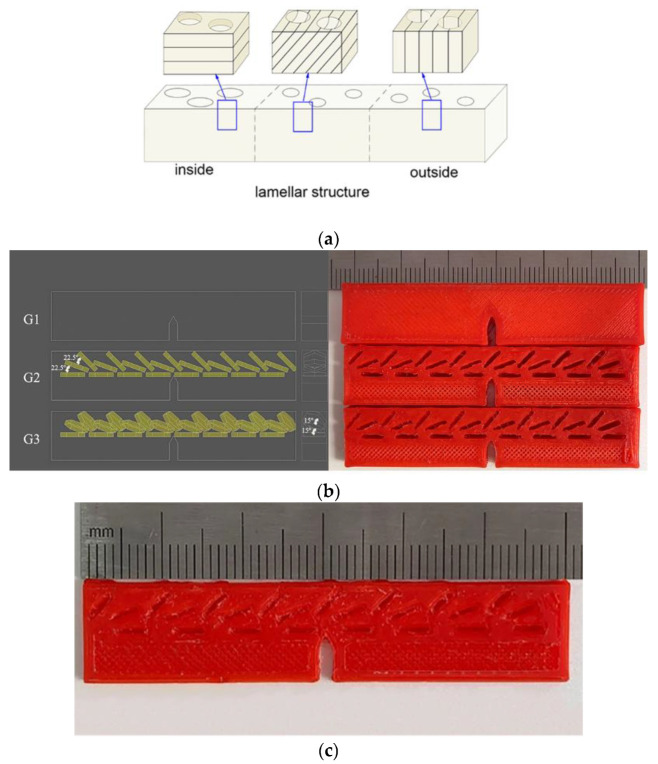
(**a**) The morphology of the bovine hoof wall. (**b**) The 3D model of the biomimetic design. (**c**) G2 specimen made by 3D printing.

The morphology structure diagram of the bovine hoof wall can be proposed as Figure 10a. Angles of the arrangement of the layers change with a deflection from 0° to 90°. Moreover, regarding the enhancement mechanism of the equine hoof wall, it has been proven that the lamellar structure (with a different arrangement) acted as stress dispersion and energy absorption, and that the tubular structure can enhance the overall performance [9]. In order to simplify the model, the tubular structures are represented by hollow hexagonal prisms, and the hollow hexagonal prisms with layer-by-layer deflection represent the different arrangement of the layers. Three groups of fracture toughness specimens are set as shown in Figure 10b. The first group of specimens (G1) has no internal structure, and the second group (G2) has three layers with the hollow hexagonal prisms arranged at 0–22.5–45°, and the third group (G3) has three layers with the hexagonal prisms arranged at 0–22.5–45° and a deflection of 15°. Figure 10c is a plan view of a 3D-printed G2 sample, clearly showing the hollow hexagonal prisms.

From Figure 10, the bovine hoof wall can be regarded as a composite material composed of a layered structure and a microtubule structure. The microtubule structure is surrounded by the materials between the tubes, and the interface forms a special interlocking structure, which increases the bonding strength of the interface. In the outer wall of the hoof wall, the layered structure is parallel to the microtubule axis, to protect the hoof wall from external material erosion; the middle layer of the hoof wall accounts for a large proportion of the total thickness of the hoof wall and the layered structure is about 30° to 60° relative to the microtubule axis. There is no obvious uniform distribution. The interface distribution of this angle makes the propagation of the crack have less resistance and plays the role of reorienting the crack. In the inner wall, the layered structure is perpendicular to the microtubule axis and supports the other layers of the bovine hoof wall. Therefore, this irregular crack propagation path will greatly lengthen the crack propagation path and improve the fracture toughness of the material.

**Figure 11:**
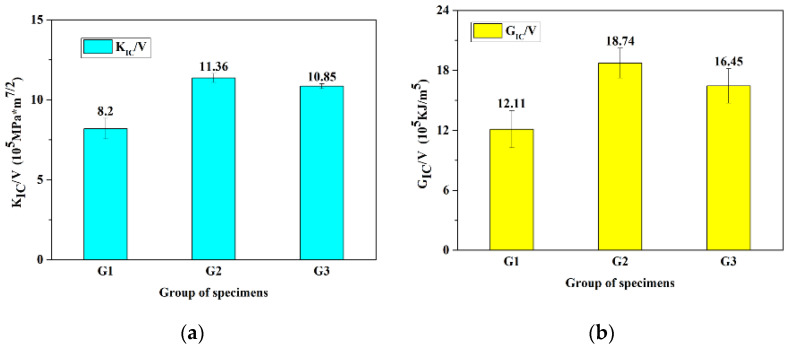
The fracture toughness-to-volume ratio of three groups of specimens. *V* is the effective volume. (**a**) *K*_IC_/*V* for different groups. (**b**) *G*_IC_/*V* for different groups.

The fracture strength of 3D-printed samples was also tested on an Instron Model 3369 universal mechanical testing machine. The key performance indicators are the critical stress intensity factor (*K*_IC_) and the critical energy release rate (*G*_IC_). The effective volume of groups G2 and G3 is smaller than that of group G1 due to the existence of hollow hexagonal prisms. Figure 11 illustrates the mechanical properties of all three groups of specimens. As for unit volume, the G2 group has the highest fracture toughness. When compared to the G1 group, it has a 39% and 55% performance improvement for *K*_IC_ and *G*_IC_, respectively. The G3 group also showed an excellent performance improvement.

## 5. Conclusions

Growth lines and tubule structures are in the bovine hoof wall, which leads to the anisotropy of mechanical properties. The LD specimens have higher reduced modulus and hardness than the TD specimens, the external parts have greater reduced modulus and hardness than the internal parts and the outside parts of the bovine hoof wall in the LD specimens have the best reduced modulus and hardness. Mechanical properties of the bovine hoof wall are sensitive to strain rates. The generalized Voigt–Kelvin model can predict the creep mechanical properties of the bovine hoof wall well.

Bovine hoof walls have a layered structure and show three fracture modes from the outside to the inside. The outer layer can form multiple interlocking interfaces that have the same effect as a multi-stage deceleration structure. In the middle layer, there are some irregular bulges and depressions along the direction of crack propagation. The fracture is very smooth in the inner layer. A hypothetical structural model of the bovine hoof wall is proposed. Compared with the samples with a uniform structure, the 3D-printed samples with a layer structure and tubules have a 39% and 55% performance improvement for *K*_IC_ and *G*_IC_, respectively.

We need high-performance materials, but also want them to have lightweight properties. Biological materials show great potential in the next generation of advanced material design. The current research provides a possible strategy for the design of protective materials of bionic structure and proposes such a structure. By combining advanced manufacturing methods with bionic structure design, a channel for a new generation of widely used advanced materials can be realized, e.g., protective materials for human bodies or automobiles.

## Figures and Tables

**Table 1:** Generalized Voigt–Kelvin model fitting parameters.

Position	NO.	*C*_0_	*C*_1_	*C*_2_	τ_1_	τ_2_	*C*_v_
TD outside	1	3024	48.66	246.20	1.69	10.25	5.69
	2	2795	137.30	16.11	4.87	1.18	10.51
	3	2798	45.42	246.90	1.84	9.55	6.21
	4	2677	35.22	122.70	1.85	6.11	8.52
	5	2698	63.05	209.30	2.27	13.02	5.35
TD middle	1	3444	303.80	55.88	8.66	1.83	12.74
	2	3418	79.50	402.10	2.57	14.38	6.02
	3	3290	17.15	197.80	1.05	5.58	13.66
	4	3527	184.10	14.86	4.80	1.09	13.70
	5	3167	6.93	108.00	6.97	2.73	21.92
TD inside	1	4072	42.38	282.60	1.51	6.18	19.62
	2	4183	130.20	117.00	16.23	2.68	40.02
	3	4069	270.80	40.19	5.56	1.40	17.87
	4	3744	429.70	116.20	15.25	2.46	7.79
	5	3273	237.70	43.66	6.73	1.67	14.59
LD outside	1	2419	9.29	120.00	0.74	4.61	7.76
	2	2228	15.95	116.40	1.03	5.11	6.78
	3	2330	178.10	34.55	9.44	1.63	4.54
	4	2434	270.90	60.92	15.71	2.20	1.81
	5	2460	152.40	34.45	7.51	1.59	5.76
LD middle	1	2780	227.50	39.37	8.47	1.76	8.60
	2	3088	8.82	156.00	0.63	4.50	11.76
	3	2573	160.30	24.91	6.18	1.38	8.39
	4	2870	136.30	5.99	4.28	0.77	10.48
	5	2747	9.15	145.00	0.77	4.75	9.77
LD inside	1	3904	604.80	99.32	17.61	2.55	7.94
	2	3242	234.80	26.25	5.59	1.48	14.33
	3	3146	17.07	197.00	1.08	5.07	12.71
	4	3039	175.20	10.20	4.84	0.70	12.88
	5	3096	10.41	179.50	0.80	4.69	12.50

## Data Availability

Data sharing not applicable. No new data were created or analyzed.
